# Hot-spots of HIV infection in Cameroon: a spatial analysis based on Demographic and Health Surveys data

**DOI:** 10.1186/s12879-022-07306-5

**Published:** 2022-04-04

**Authors:** Arsène Brunelle Sandie, Jules Brice Tchatchueng Mbougua, Anne Esther Njom Nlend, Sokhna Thiam, Betrand Fesuh Nono, Ndèye Awa Fall, Diarra Bousso Senghor, El Hadji Malick Sylla, Cheikh Mbacké Faye

**Affiliations:** 1 African Population and Health Research Center - West Africa Regional Office, Dakar, Senegal; 2 Centre Pasteur du Cameroun - Service d’Epidémiologie et de Santé Publique, Yaoundé, Cameroon; 3 Health Ebene Consulting, Yaoundé, Cameroon; 4 Ecole Nationale Supérieure Polytechnique de Yaoundé, Université de Yaoundé I, Yaoundé, Cameroon

**Keywords:** Cameroon, Clustering, HIV, Getis–Ord statistics, Hot-spots, Spatial

## Abstract

**Background:**

The Human Immunodeficiency Virus(HIV) infection prevalence in Cameroon has decreased from $5.28\%$ in 2004 to $2.8\%$ in 2018. However, this decrease in prevalence does not show disparities especially in terms of spatial or geographical pattern. Efficient control and fight against HIV infection may require targeting hotspot areas. This study aims at presenting a cartography of HIV infection situation in Cameroon using the 2004, 2011 and 2018 Demographic and Health Survey data, and investigating whether there exist spatial patterns of the disease, may help to detect hot-spots.

**Methods:**

HIV biomarkers data and Global Positioning System (GPS) location data were obtained from the Cameroon 2004, 2011, and 2018 Demographic and Health Survey (DHS) after an approved request from the MEASURES Demographic and Health Survey Program. HIV prevalence was estimated for each sampled area. The Moran’s I (MI) test was used to assess spatial autocorrelation. Spatial interpolation was further performed to estimate the prevalence in all surface points. Hot-spots were identified based on Getis–Ord (Gi*) spatial statistics. Data analyses were done in the R software(version 4.1.2), while Arcgis Pro software tools’ were used for all spatial analyses.

**Results:**

Generally, spatial autocorrelation of HIV infection in Cameroon was observed across the three time periods of 2004 ($MI=0.84$, $p-value < 0.001$), 2011 ($MI=0.80$, $p-value < 0.001$) and 2018 ($MI=0.87$, $p-value < 0.001$). Subdivisions in which one could find persistent hot-spots for at least two periods including the last period 2018 included: Mbéré, Lom et Djerem, Kadey, Boumba et Ngoko, Haute Sanaga, Nyong et Mfoumou, Nyong et So’o Haut Nyong, Dja et Lobo, Mvila, Vallée du Ntem, Océan, Nyong et Kellé, Sanaga Maritime, Menchum, Dounga Mantung, Boyo, Mezam and Momo. However, Faro et Déo emerged only in 2018 as a subdivision with HIV infection hot-spots.

**Conclusion:**

Despite the decrease in HIV epidemiology in Cameroon, this study has shown that there are spatial patterns for HIV infection in Cameroon and possible hot-spots have been identified. In its effort to eliminate HIV infection by 2030 in Cameroon, the public health policies may consider these detected HIV hot-spots, while maintaining effective control in other parts of the country.

## Introduction

In recent years, the national and international communities have scaled up strategies to control HIV/AIDS. The Millennium Development Goal(MDG) of the United Nations has integrated the fight against HIV/AiDS in goal 6. Many countries adopted the MDG and as a result, according to World Health Organisation(WHO), HIV new infections declined by $38\%$ between 2001 and 2013 worldwide [1]. In the Sustainable Development Goals (SDG), the fight against HIV was integrated into goal 3.3, where the main goal was to end the AIDS epidemics, tuberculosis, malaria, neglected tropical diseases, and combat hepatitis, water-borne diseases, and other communicable diseases by 2030. To reach this goal, the United Nations AIDS (UNAIDS) launched and advocated the 90-90-90 targets. These targets were to be reached by 2020 and consisted of: $90\%$ of all the people living with HIV being able to know their HIV status, $90\%$ of all people with diagnosed HIV infection will receive sustained antiretroviral therapy and $90\%$ of all people receiving antiretroviral therapy will have viral suppression [2]. With the efforts of most countries worldwide, new infections of HIV globally declined by about $23\%$ between 2010 and 2019 [3].

Cameroon had also experienced a bright situation in terms of declination of HIV infection. In fact, from 2011 to 2018, the prevalence of HIV infection in Cameroon declined from $5.55\%$ to $2.8\%$ [4, 5]. However, there were persistent disparities in HIV infection in Cameroon in terms of age, place of residence, region and gender. Prevalence was higher in the female population ($3.5\%$, versus $1.9\%$ in male population), urban areas ($2.9\%$, versus $2.4\%$ in rural), and in adults aged 35-39 years. In terms of regional disparities, it was found that HIV infection was more prevalent in the South ($5.8\%$), East ($5.6\%$), Adamawa ($4.1\%$), North-West ($4.0\%$), and Center ($3.5\%$) regions. These regions were defined in the 2018-2022 National Strategic Plan for HIV/AIDS and STIs as priority areas of intervention. Moreover, these regions were to be targeted more in the fighting against HIV infection in Cameroon [6]. However, regions are the first level of geographical and administrative division in Cameroon. They are generally very large with heterogeneous populations. For efficient interventions for HIV elimination, it would therefore be relevant to target hot-spots. These are accurate spots areas where the infection gets to spread the most. This study mainly aimed to identify the HIV infection hot-spots clusters in Cameroun for the periods, 2004, 2011, and 2018. Identifying hot-spots for diseases is important for public health authorities who should adopt them for better-targeted interventions. This has been done in other settings such as in Mongolia [7], Ethiopia [8], Brazil [9], Shanghai [10], Malawi [11], Nigeria [12] and India [13, 14].

## Methods

### Study design and sample

Demographic and Health Surveys are on a nationwide scale, periodic, and generally based on similar methodologies in sampling surveys. In Cameroon, it has been performed for the periods 1991, 1998, 2004, 2011, and 2018. They were cross-sectional surveys, where individuals living in ordinary households were targeted. Basically, the Cameroun DHS was based on two-stage stratified random sampling. In the first stage, the enumeration areas (EA) from the general census of the population were sampled proportionally to the number of households in clusters after stratification in rural and urban EA respectively. Then, at stage 2, the households were sampled within the sampled EA in the first stage systematically and with equal probability. Finally, in the half of the total obtained in the sample, all 15-64 years old men and women were eligible for HIV screening tests. Table 1 shows the sample population for respective DHS periods in Cameroon. More details about the sample survey design of different Cameroon DHS could be obtained in reports [4, 5, 15].

**Table 1:** Sample sizes and HIV prevalences for 2004, 2011 and 2018

Periods	Sample sizes	Prevalence
2004	10194	$5.28\%$
2011	14442	$5.55\%$
2018	14085	$2.80\%$

### Data sources and measurements

After an approved request from the DHS program, the GPS and HIV biomarkers data were downloaded from their website (http://www.dhs.program.com). For the periods of 1991 and 1998, GPS and HIV biomarkers data were not collected. Therefore, in this study, only the periods of 2004, 2011 and 2018 were considered for the spatial analysis of HIV infection in Cameroon. Blood samples were screened and double-checked for positive cases by the Pasteur Center of Cameroon. Then, for quality assessment, screened samples were re-screened externally by the Chantal Biya International Reference Center. A concordance of 98.96% was found between the outcomes of both centers. The primary endpoint of this study was confirmed HIV positive cases in the 15-64 age group. Table 1 shows the HIV prevalence for the respective periods.

An important feature in the DHS surveys is the presence of georeferenced data. Coordinates of clusters were observed on the field using GPS receivers. For confidential purposes, the positions of sampled locations were randomly displaced according to the type of area :

- In urban clusters, the displacement was done with a radius from 0 to 2 kilometers.
- In rural clusters, the displacement was done with a radius from 0 to 5 kilometers, with a further 1% of the rural clusters displaced a minimum of 0 and a maximum of 10 kilometers.

Further details about the GPS data, its collection and processing in the DHS surveys can be found in [16].

### Statistical methods and maps tools

*Moran I test :* Introduced by [17], the Moran I test is the global test most commonly used for assessing spatial autocorrelation. The null hypothesis is that the spatial distribution of the studied phenomenom is random, versus the alternative hypothesis in which the studied phenomenom is not random (there is a spatial autocorrelation). Moran I test is based on a neighborhood matrix that specifies the link between spatial units. In this study, the k nearest neighbor matrix, which for each spatial unit determines the k-nearest neighbors based on the distance between them (the default number of neighbors adopted during the analysis was 8). A significant Moran I test indicates that there is a presence of spatial autocorrelation, but could not identify the hot or cold spots areas. The Arcgis Pro software was further used to present a cartography of HIV infection in Cameroon for the respective periods of 2004, 2011, and 2018.

*Spatial interpolation :* In DHS surveys, the spatial unit were sampled EA, therefore, the HIV prevalence at spatial points of the territories which were not sampled are unknown. Spatial interpolation is a statistical technique which allows to estimate the unobserved attribute at the non-sampled EAs from the attributes of sampled EAs. In such way, the HIV prevalence estimate at each point of the surface can be obtained. Spatial interpolation is generally applied on the assumption of spatial autocorrelation. After assessing for spatial autocorrelation, the spatial interpolation based on inverse distance weighting (IDW) was applied to obtain the HIV prevalence in all surface points.

*Hot-spots identification :* The determination of hot-spot clusters was based on Getis-Ord statistics introduced by [18]. The $z-score$ of the prevalence of each feature were estimated, the idea was to compare each $z-score$ to all others. To be a statistically significant hot spot, a feature will have a high value and be surrounded by other features with high values as well. This method is implemented in the Arcgis Pro software through the Spatial Statistics Tools. In this work, the optimzed hot-spot analysis was used, as it automatically aggregates prevalence data, identifies an appropriate scale of analysis, and corrects for both multiple testing and spatial dependence.

**Fig. 1:**
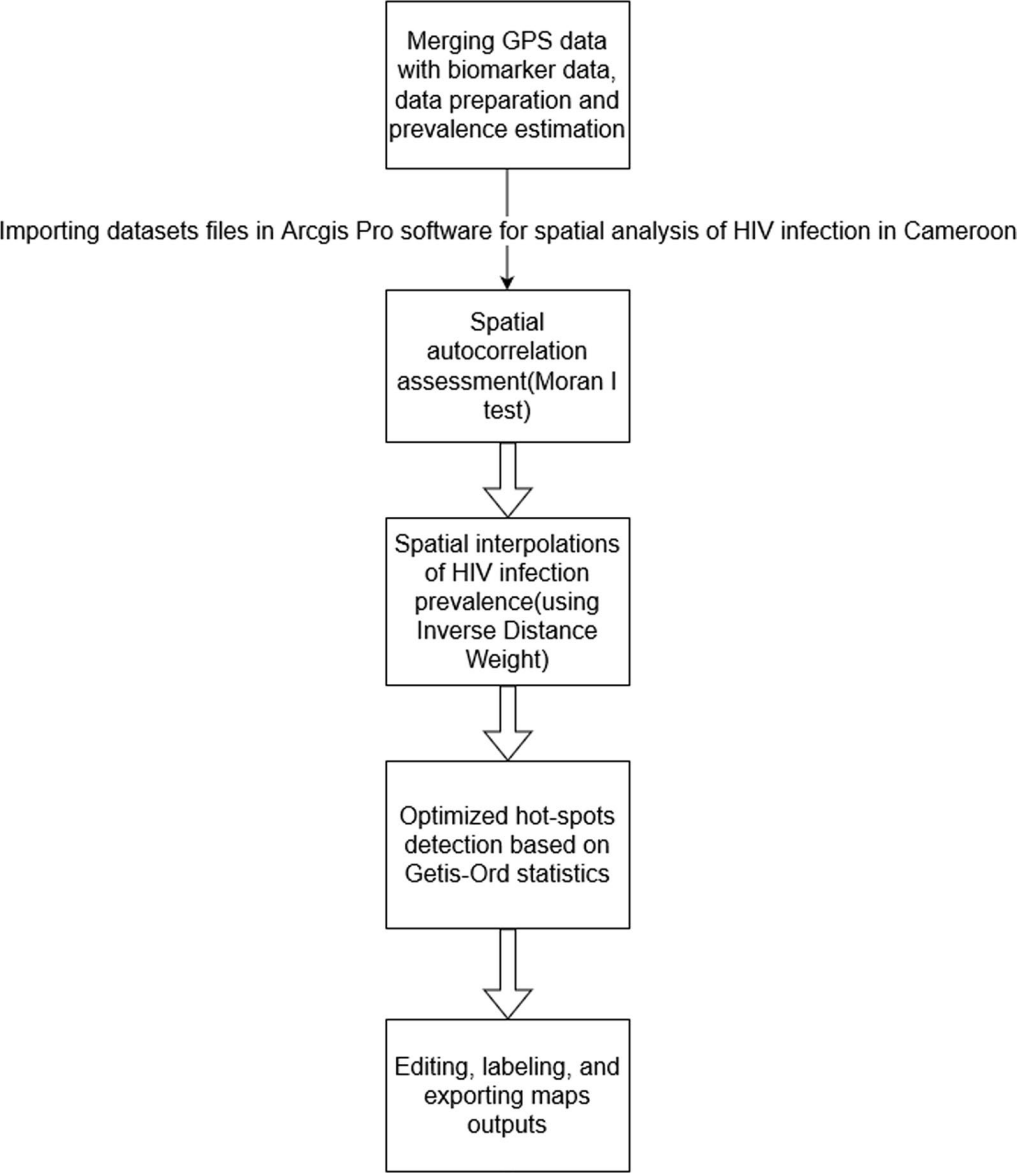
Flow diagram of the methodology process

Figure 1 summarizes the methodology used in the spatial analysis of HIV infection in Cameroon in 2004, 2011, and 2018.

## Results

Table 1 shows HIV prevalence in Cameroun in 2004, 2011 and 2018 periods. Basically, the HIV prevalence in Cameroon was $5.28\%$, $5.55\%$ and $2.80\%$ for the periods of 2004, 2011 and 2018 respectively. Hence, from 2004 to 2011, there was a relative increase of $5.11\%$, and from 2011 to 2018, the epidemiology of HIV infection decreased by $49.55\%$.

### Spatial auto-correlation of HIV infection in Cameroon

**Fig. 2:**
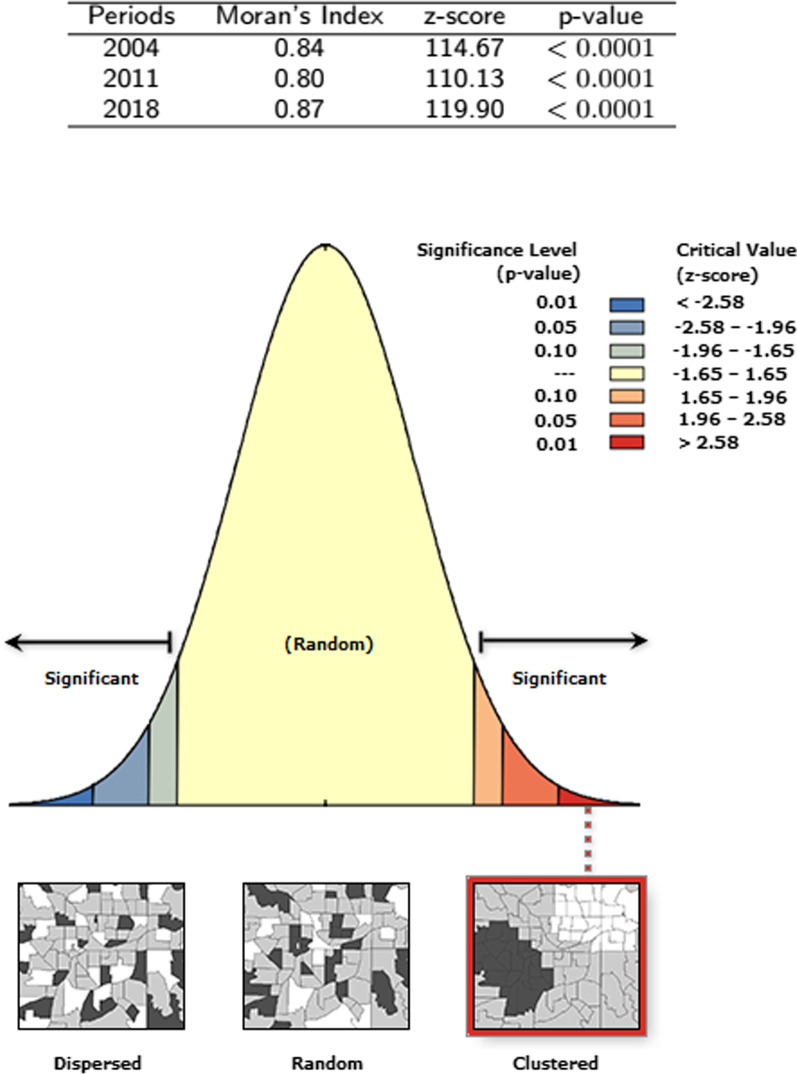
Moran’s spatial auto-correlation test statistics outputs for HIV infection in Cameroon for the periods 2004, 2011 and 2018

The outputs of spatial auto-correlation of HIV infection in Cameroon are summarized in Fig. 2 for the periods 2004, 2011 and 2018 respectively. Spatial autocorrelation of HIV infection in Cameroon for each of the periods 2004, 2011 and 2018 was observed ($p-value<0.0001$). The Moran’s Indices were 0.84, 0.80, and 0.87 respectively. Meaning that for the three periods, there were spatial patterns of HIV infection in Cameroon.

### Hot-spots clusters of HIV infection in Cameroon

**Table 2:** Subdivisions of significant hot-spots clusters of HIV infection in Cameroon in 2004, 2011 and 2018 respectively

Periods	Subdivisions
2004	Vina, Mbéré, Djérem, Lom et Djérem,
	Boumba et Ngoko, Mbam et Kim, Haut Nyong,
	Nyong et Mfoumou, Mefou et Afamba, Mfoundi,
	Dja et Lobo, Océan, Mvila, Nyong et So’o,
	Sanaga Maritime, Wouri, Fako, Moungo, Meme,
	Kupe-Manenguba, Ndian, Bamboutos, Ngo-Ketunjia,
	Mezam, Momo, Manuyi, Boyo, Bui, Menchun,
	Donga Mantung, Mayo-Bayo.
2011	Vina, Mbéré, Djérem, Lom et Djérem, Kadey
	Boumba et Ngoko, Haute Sanaga, Mbam et Kim
	Nyong et Mfoumou, Haut Nyong, Dja et Lobo, Mvila
	Nyong et So’o, Nyong et Kellé, Vallée du Ntem
	Océan, Meme, Moungo, Fako, Bui, Boyo
2018	Faro et Déo, Mbéré, Lom et Djerem, Kadey,
	Boumba et Ngoko, Haute Sanaga, Nyong et Mfoumou,
	Nyong et So’o Haut Nyong, Dja et Lobo, Mvila,
	Vallée du Ntem, Océan, Nyong et Kellé,
	Sanaga Maritime, Menchum, Donga Mantung, Boyo,
	Mezam and Momo.

**Fig. 3:**
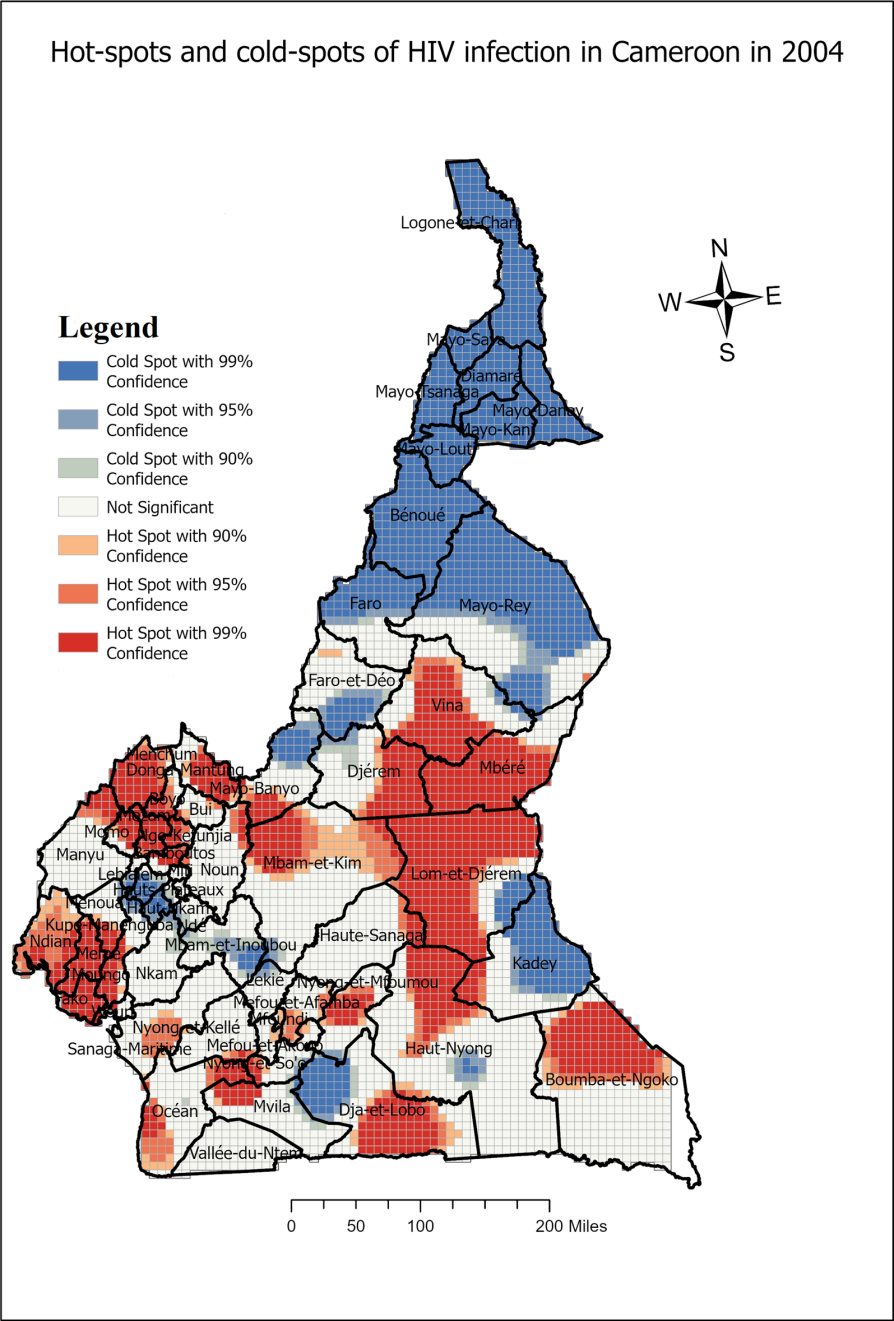
Spatial distribution of HIV infection in Cameroon and hot-spots clusters 2004

**Fig. 4:**
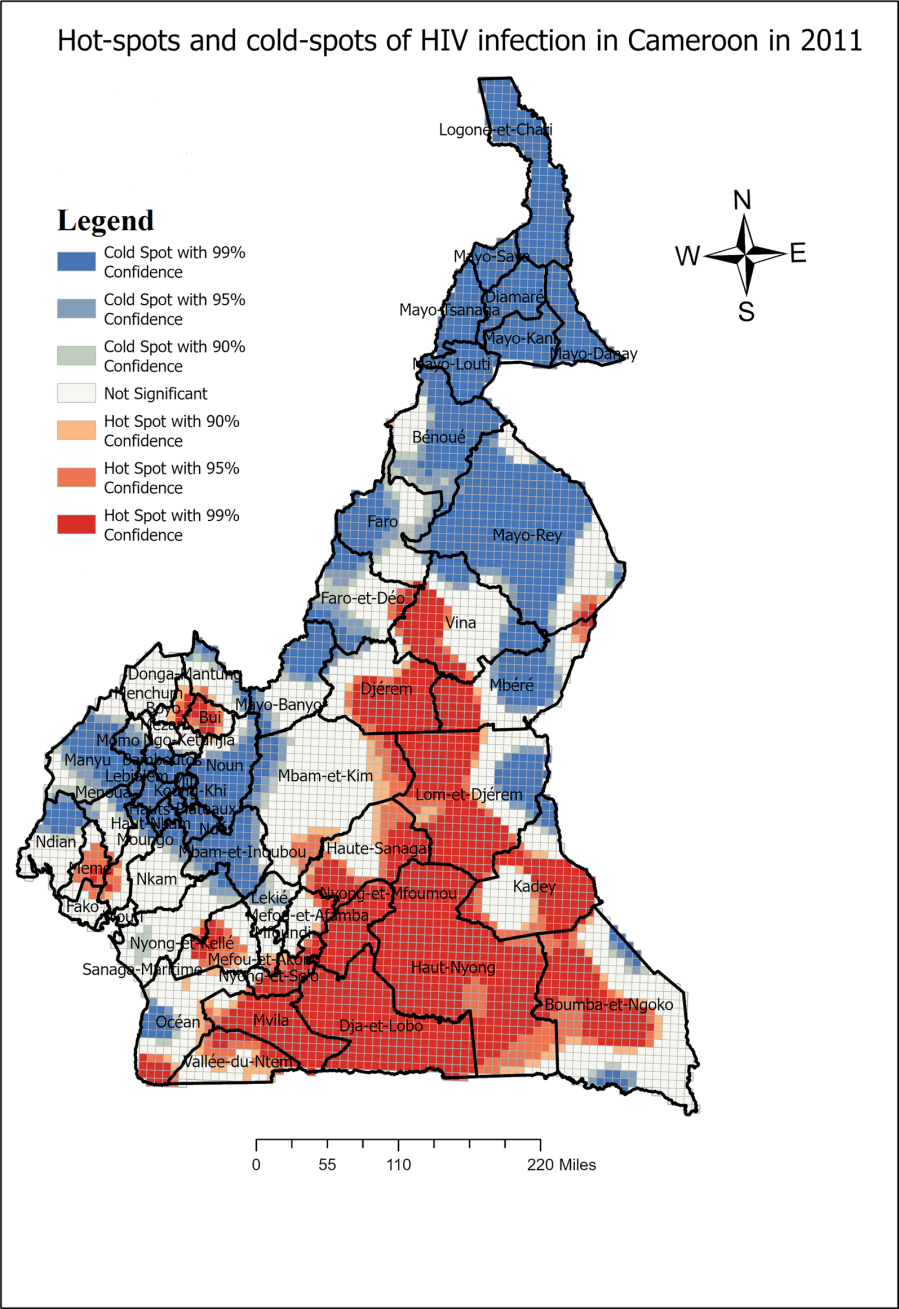
Spatial distribution of HIV infection in Cameroon and hot-spots clusters 2011

**Fig. 5:**
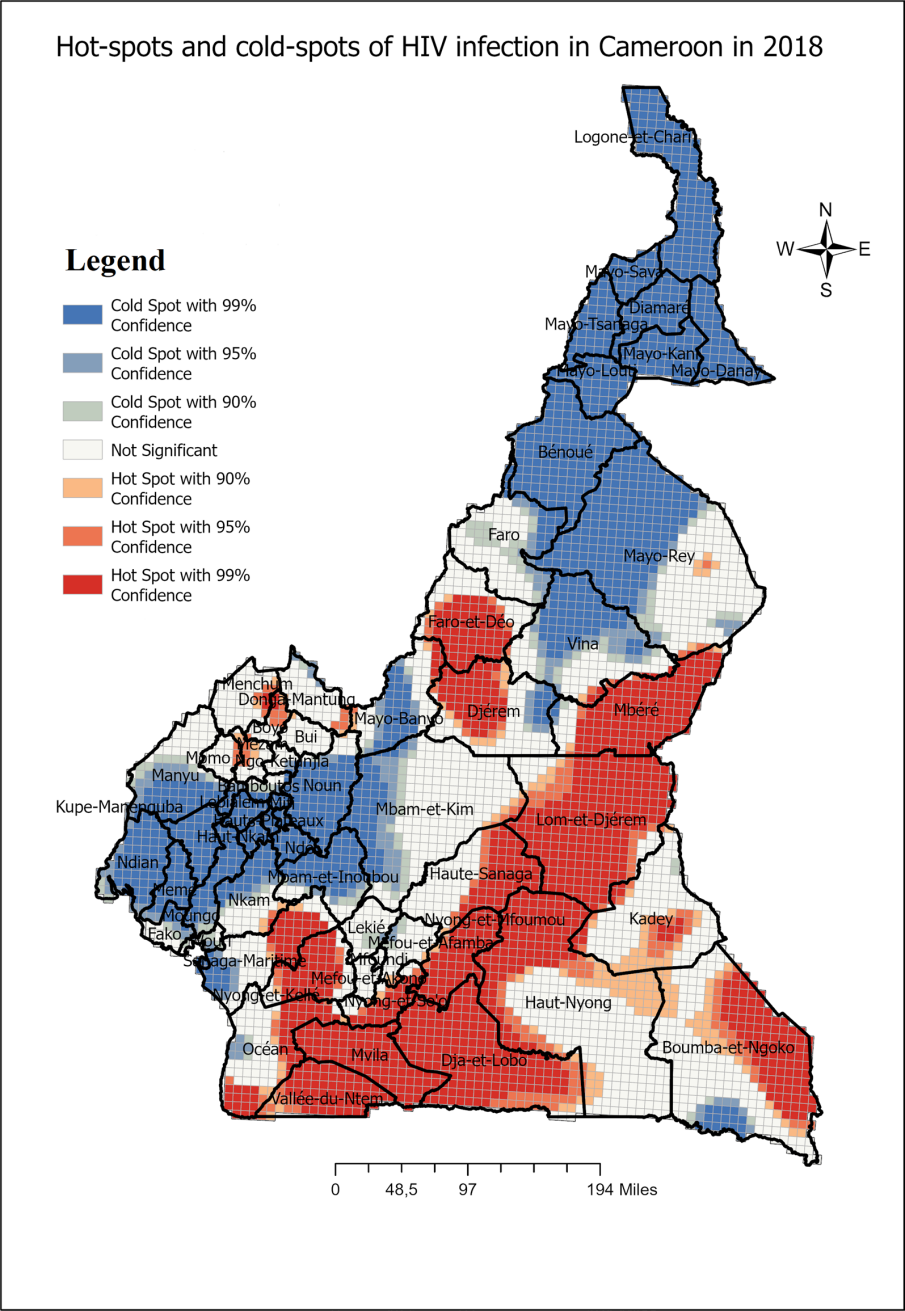
Spatial distribution of HIV infection in Cameroon and hot-spots clusters 2018

Table 2 displays the subdivisions of HIV infection hot-spots in Cameroon for the periods 2004, 2011 and 2018 respectively, while, Figs. 3,  4, and 5 display the maps of hot-spots of HIV infection in Cameroon for the respective periods.

One can define persistent subdivisions with HIV infection hot-spots, subdivisions in which hot-spots were detected in at least the last two periods including the 2018 period. These subdivisions include : Mbéré, Lom et Djerem, Kadey, Boumba et Ngoko, Haute Sanaga, Nyong et Mfoumou, Nyong et So’o Haut Nyong, Dja et Lobo, Mvila, Vallée du Ntem, Océan, Nyong et Kellé, Sanaga Maritime, Menchum, Dounga Mantung, Boyo, Mezam and Momo. Faro et Déo emerged only in 2018 as a subdivision with HIV infection hot-spots. However, Mfoundi, Vina, Wouri, Meme, Moungo, Kupe-Manenguba, Ndian, Bamboutos, Fako, Ngo-Ketunjia, Mayo-Bayo, Manuyi, Bui stopped hosting HIV infection hot-spots from either 2004 or 2011 to 2018.

## Discussion

This study has provided a spatial analysis of HIV infection situation in Cameroon for the periods of 2004, 2011 and 2018, while determining the hot-spots. These analyses go beyond the regional analysis which is commonly adopted by public health policies in Cameroon. In fact, in the recent document of the National Strategic Plan for HIV/AIDS, 6 regions; Adamaoua, East, North-West, Center, South-West and Littoral were identified as priority areas of intervention [6]. A study by [19] revealed that HIV risk among pregnant women in Cameroon was higher in the East, North-West, and South-West regions. However, the exact localities or subdivisions in these regions were not identified. In such a way, the interventions on a large scale such as regions may be less efficient. The spatial analysis in this study aims at predicting hot-spots of HIV infection for the periods of 2004, 2011 and 2018, with the included localities. Based on the predicted hot-spots, the current regions which needed to be prioritize should be : Adamaoua, East, Center, South and North-West. We remark that the predicted hot-spots overlapp with the targeted regions in the strategic document for the fight against AIDS in Cameroon, our study identified the subdivisions with the hot-spots of HIV infection in Cameroon.

It has been found that the subdivisions: Nyong et Mfoumou, Haute Sanaga, Lom et Djerem are in the mid-way of the corridor Douala-Bangui. This stretch is among the longest and the most densely populated in the country which goes up to the capital city of Central Africa Republic (CAR). Cities and towns within these subdivisions have truck stops and rest-spots for truck drivers. Therefore these subdivisions on the corridor are hot-spots clusters of HIV infection as found in other settings [8, 11, 12]. In fact, truck drivers generally have high sexual risk behaviors, they practice unprotected sexual intercourse with multiple partners living in rest-spots cities [20–23]. Another characteristic of some hot-spots was that most of them were found on the cross-borders of Cameroon with CAR (Mbere, Lom et Djerem, Kadey, Boumba et Ngoko) and Congo (Haut-Nyong, Océan, Dja et Lobo and Mvila) respectively. Some studies revealed that border areas are generally subjected to a high rate of unsafe sex practice and a low level of HIV-related knowledge, attitudes, and practices [24–26]. This may explain the high risk of HIV infection in these subdivisions on the borders where there is high human mobility in and out. Other hot-spot subdivisions, may particularly experience sexual risk behaviors especially in terms of condom non-use. That may be the case in the Sanaga-Maritime subdivision where a study on adolescents in Edéa (Capital city of Sanaga-Maritime) found that adolescents both males and females had poor condom use perception [27].

One finds that scientific works on contextual and sociocultural factors of HIV infection in Cameroon are limited. Particularly, studies at the subdivisions levels are almost inexistent. The investigation of HIV infection with the associated contextual factors at the subdivision level could be relevant for the characterization of identified hot-spots of HIV infection in Cameroon. This could constitute a topic for further studies.

## Conclusions

HIV infection has significantly decreased in Cameroon from 2011 to 2018. Cameroonian public health authorities usually based their policies on regional disparities analysis. Beyond these regional analyses, this study performed a finer analysis which uses geographical coordinates to identify hot-spots areas. However, a spatial analysis in this study has indicated that there remain persistent HIV infection hot-spots. This study has provided mappings of these hot-spots. Therefore, subdivisions and localities which need more attention are now identified. For an efficient fight against HIV infection in Cameroon, public health policies should target more the identified hot-spots with adapted interventions. However, the surveillance and control in other areas which were not identified as hot-spot clusters should continue.

## Data Availability

The data that support the findings of this study are available from the DHS program but restrictions apply to the availability of these data, which were used under license for the current study, and so are not publicly available. Data are however available from the authors upon reasonable request and with permission of DHS program.

## Abbreviations

HIV: Human immunodeficiency virus
AIDS: Acquired Immuno Deficiency Syndrome
EA: Enumeration area
DHS: Demographic and Health Survey
GPS: Global positioning system
STI: Sexually transmitted infection
WHO: World Health Organization
UNAIDS: United Nations Programme on HIV/AIDS
CAR: Central Africa Republic
